# Axillobifemoral bypass for total abdominal occlusion secondary to Takayasu’s arteritis: A case report

**DOI:** 10.1016/j.ijscr.2019.07.031

**Published:** 2019-07-19

**Authors:** Omar Jiménez-Zarazúa, Lourdes Noemí Vélez-Ramírez, María Andrea Martínez-Rivera, Abraham Hernández-Ramírez, Pascual Palomares-Anda, María Alcocer-León, Angélica Monserrat Becerra-Baeza, Jaime D. Mondragón

**Affiliations:** a Hospital General León, Department of Internal Medicine, Mexico; b Universidad de Guanajuato, Department of Medicine and Nutrition, Mexico; c Hospital General León, Department of Radiology, Mexico; d Hospital General León, Department of Hematology, Mexico; e Hospital Regional ISSSTE León, Department of Internal Medicine, Mexico; f University of Groningen, University Medical Center Groningen, Department of Neurology, the Netherlands; g University of Groningen, University Medical Center Groningen, Alzheimer Research Center, the Netherlands

**Keywords:** Axillobifemoral bypass, Case report, Takayasu’s arteritis, Total abdominal occlusion

## Abstract

**Introduction:**

Takayasu’s arteritis (TA) is a rare disease that mainly affects large arteries. Approximately 20% of TA patients will require surgical intervention secondary to arterial complications such as intermittent claudication, persistent hypertension refractory to treatment, and heart failure.

**Presentation of case:**

The case of a 22-year-old female with TA of five years of evolution is presented. The patient deteriorated clinically after five years of corticosteroid and immunosuppressant management requiring surgical intervention with an axillobifemoral bypass for a total abdominal occlusion. Onset, pre-surgical and post-surgical Doppler ultrasonography as well as abdominal angiotomographies document and corroborate the patient’s clinical and hemodynamic improvement.

**Discussion:**

Very limited literature exists regarding surgical interventions for TA patients. While most reported cases present an endovascular surgical management. Open surgical procedures have lower rates of restenosis than endovascular management. Although endovascular management is less invasive than extra-anatomical axillobifemoral bypass, the patient was not a candidate for endovascular stent graft placement due to the increased risk for vascular injury and subsequent perforation. Approximately a fifth of TA patients are candidates for surgical intervention over time.

**Conclusion:**

Vascular surgery in TA cases becomes an option when the patient does not improve clinically after administration of medical treatment. Although endovascular management has fewer complications, the rate of restenosis is higher. Patients at risk of restenosis and who have increased perioperative vascular risk can benefit from open surgical procedures. Surgical management should be tailored to the patient’s needs.

## Highlights

• Vascular surgery becomes an option when the TA patient does not improve clinically.
• Open surgical procedures have lower restenosis rates than endovascular management.
• Patients at risk of restenosis can benefit from open surgical procedures.
• Patients with increased perioperative vascular risk can benefit from open surgery.
• Surgical management should be tailored to the patient’s needs.

## 1 Introduction

Takayasu’s arteritis (TA) is a chronic, inflammatory, granulomatous, idiopathic disease that affects arteries such as the aorta and its ramifications, as well as the pulmonary artery [1,2]. Arterial vasculitis in TA is characterized by both dilation and stenosis. Histopathological findings include a panarteritis which shows inflammation around the vasorum vessel as well as the adventitia, with a perivascular mononuclear infiltrate, composed mainly of CD4+/CD8+ lymphocytes, plasma cells, and macrophages [1]. TA is a rare disease and it is more prevalent in Central and South America, Africa, India, and the Far East [3]. TA has a prevalence in the Japanese population of 0.004% and a yearly incidence of between 0.7–4.7 cases per million [[3], [4], [5], [6]].

The clinical presentation associated with TA is unspecific. Symptoms can range from constitutional symptoms (e.g. fatigue, fever, and weight loss) to claudication, cephalalgia, syncope, angina pectoris, and abdominal pain which are associated to vascular territory affected by the arteritis [6,7]. Although there is no imaging or laboratory gold standards with adequate sensitivity of specificity for TA, the American College of Rheumatology and Ishikawa diagnostic criteria are the most widely adopted criteria [[7], [8], [9]]. These criteria incorporate age at disease onset, muscular and arterial tenderness, claudication, pulse characteristics, blood pressure differences, findings related to arterial auscultation, arteriogram findings, and echocardiographic alterations [8,9]. While 20% of TA patients have a self-limited disease progression, up to 20% will require surgical intervention secondary to arterial complications [7]. Intermittent claudication, persistent hypertension refractory to treatment, and heart failure are among the most common indications for surgical intervention in TA [10].

We present the case report in line with the SCARE criteria [11] of a patient with TA treated in a university hospital, who after five years of corticosteroid and immunosuppressant management deteriorated clinically requiring surgical intervention with an axillobifemoral bypass for a total abdominal occlusion. Very limited literature exists regarding surgical interventions for TA patients. This case contributes to the existing literature with a successful surgical case that documented through various imaging modalities a patient for over five years.

## 2 Case presentation

A 22-year-old female arrived at the Emergency Department presenting claudication when walking less than 300 m as well as increased paresthesia and dysesthesia in both pelvic limbs. The claudication, paresthesia, and dysesthesia began five years earlier. At the time of onset, the claudication was bilateral after walking approximately 1500 m with improvement after rest, while the paresthesia and dysesthesia were bilateral and involved all four extremities. At that time, the patient was diagnosed with Takayasu’s arteritis (TA) by the Rheumatology department from this institution based on immunological profile (i.e. rheumatoid factor, antinuclear antibodies, anticardiolipin antibodies, and antineutrophil cytoplasmic antibodies (ANCA), the American College of Rheumatology and Ishikawa criteria. [8,9] Six months prior to the patient’s visit to this hospital, claudication progressed (i.e. reduction in the distance able to walk to 500 m, increasing paresthesia and dysesthesia frequency). The patient had no relevant family and personal non-pathological history to her current condition. The patient denied the use of controlled substances, allergies, past blood transfusions, traveling to regions with endemic diseases within the last three months, tattoos and body piercings.

Upon initial physical examination, we found a patient recumbent with freely chosen body position, Glasgow coma score of 15, without focal neurologic deficits nor meningeal sings, aware of his environment, with reference to place, time, and people. The patient’s integumentary system was hydrated and without alterations, while the head and neck exploration had no alterations. Upon inspection, palpation, and percussion the cardio-respiratory system and abdomen had no abnormal findings. Precordial auscultation revealed tachycardia, but no aggregate phenomena. Abdominal auscultation revealed a systolic murmur grade III/IV at the mesogastrium. Right upper limb exploration revealed normal axillar, humeral, and radial pulses (i.e. presence of rhythmic, with normal intensity +++/+++, normal amplitude, and having a synchronous frequency with heart rate). The right ulnar artery pulse was absent. Upon left upper limb exploration, palpation showed the presence of normal axillar and humeral pulses; while radial and ulnar pulses were absent. Lower limb exploration showed absence of bilateral femoral, popliteal and posterior tibial pulses. The skin presented cyanotic appearance, especially of the toes of both feet. Upon palpation, reduced skin temperature was noticed and absence of edema. Upon admission, the patient had the following vital signs: blood pressure 100/70 mmHg in the right arm, 80/60 mmHg in the left arm, blood pressure in the right leg and the left leg were not detectable; heart rate 85bpm; respiratory rate 17 rpm; temperature 36 °C; weight 65 kg; height 167 cm; body mass index23.3 kg/m [2]. Laboratory results at admission are presented in Table 1 and the follow-up laboratory results in Table 2.

**Table 1:** Laboratory test results upon admission the Emergency Department.

Full Blood Count	
Hemoglobin at admission	14.5 g/dL
Hematocrit	44.5%
Erythrocyte count	5300 μL
Platelet count	296,000μL
Mean corpuscular volume	85fL
Mean corpuscular hemoglobin concentration	27.30 g/dL
Leukocyte count	9200μL
Lymphocytes	18.8%
Neutrophils	69.9%
Monocytes	9.1%
Eosinophils	2.1%
Basophils	0.1%
Blood Chemistry	
Glucose	88 mg/dL
Albumin	2.42 gr/dL
Urea nitrogen	0.60 mg/dL
Blood urea nitrogen	12.9 mg/dL
Uric acid	3.6 mg/dL
Cholesterol	130 mg/dL
Triglycerides	140 mg/dL
Liver Function Enzymes	
Aspartate transaminase	9 U/L
Alanine transaminase	12 U/L
Lactate dehydrogenase	10 U/L
Albumin	3.5 mg/dL
Alkaline phosphatase	66.8 U/L
Gamma-glutamyl transpeptidase	10 U/L
Blood Coagulation	
Prothrombine time	18 Sec
Partial thromboplastin time	40 Sec
International normalized ratio	1.36
Electrolytes	
Sodium	mEq/dL
Potassium	mE/dL
Chlorine	mEq/dL
Calcium	mg/dL
Phosphorus	mg/dL
Magnesium	mEq/dL

**Table 2:** Complementary laboratory test results.

Follow-up	
Immunological assay	
Anti-double-stranded deoxyribonucleic acid	0.9 UI/mL
Anti-cardiolipin IgG	1.0 UI/mL
Anti-cardiolipin IgM antibody	3.0 UI/mL
Erythrocyte sedimentation rate	15 mm/h
C-reactive protein	3.80 mg/dL
Viral assay	
Hepatitis B virus	Negative
Hepatitis C virus	Negative
Human immunodeficiency virus	Negative
Urinalysis	
Appearance	Crystalline
pH	6.5
Specific gravity	1.020
Proteins	30 mg/dL
Ketones, glucose, and nitrite	Negative
Leukocytes	2 per high power field
Erythrocytes	3 per high power field
Bacteria	Absent

## 3 Clinical history

During initial symptom onset (i.e. five years prior to this hospital admission) the patient had absent left upper limb distal pulses (i.e. radial and ulnar pulse). The right upper limb pulses (i.e. axillar, humeral, ulnar, and radial) and left axillar and humeral pulses had were present, rhythmic, with normal intensity (i.e. +++/+++), and having a synchronous frequency with heart rate. Lower limb exploration showed normal left limb pulses (i.e. femoral, popliteal, posterior tibial and dorsal pedis) and decreased right femoral (i.e. present, decreased frequency and amplitude, and intensity ++/+++), right popliteal (i.e. present, rhythmic, intensity +/+++, and decreased amplitude) pulses and absent right limb distal pulses (i.e. posterior tibial and dorsalis pedis).

**Fig. 1:**
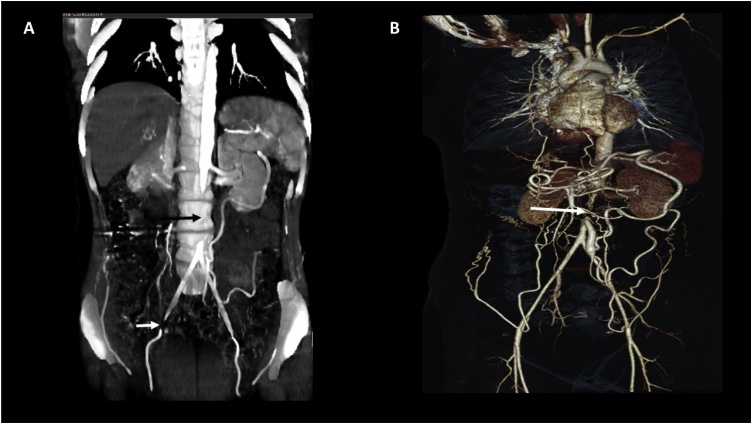
Abdominal angiotomography showing aortic and multiple vessel stenoses at the time of diagnosis. A) Coronal reconstruction showing emergence of aortic artery 27 mm from the renal arteries with stenosis superior to 50%. The length of the stenosis of approximately 64 mm (marked by arrow). B) Coronal 3-D reconstruction. Multiple collateral arteries are present with a prominent and dilated arc of Riolan with vascular redistribution to the iliac arteries. Stenosis marked by arrow.

During the initial onset, the patient underwent pulsed-wave Doppler ultrasonography (i.e. spectral) of the lower limbs. The reported arterial blood flow velocities of the common femoral artery, right superficial femoral artery, left superficial femoral artery, right popliteal artery, and left popliteal artery were diminished and are found in Table 3. The waveform was monophasic with a dampened pattern compatible with a bilateral femoropopliteal insufficiency. Doppler ultrasonography of the abdominal aorta had a 50% diameter reduction below the renal arteries level. An abdominal angiotomography reported aortic and multiple vessel stenoses (Fig. 1a).

**Table 3:** Blood flow velocities at time of diagnosis.

Anatomical region	Velocity (cm/sec)	
Upper limbs	Right	Left
Axillary artery	77	87
Proximal segment brachial artery	76	82
Middle segment brachial artery	70	75
Distal segment brachial artery	62	51
Proximal segment radial artery	45	37
Distal segment radial artery	31	35
Proximal segment ulnar artery	43	33
Distal segment ulnar artery	42	33
Abdominal aorta		
Suprarenal segment	160	
Infrarrenal proximal segment	253	
Infrarrenal distal segment	288	
External iliac artery	230	153
Lower limbs	Right	Left
Common femoral artery	36	25
Proximal segment superficial femoral artery	21	16
Middle segment superficial femoral artery	17	15
Distal segment superficial femoral artery	13	11
Popliteal artery	12	11
Proximal segment anterior tibial artery	12	19
Middle segment anterior tibial artery	12	6
Distal segment anterior tibial artery	7	7
Proximal segment posterior tibial artery	10	10
Middle segment posterior tibial artery	8	6
Distal segment posterior tibial artery	10	8
Proximal segment fibular artery	7	13
Middle segment fibular artery	6	7
Distal segment fibular artery	6	6

Initial treatment was prednisone 50 mg orally (*per os*, PO) every (*quaque*, q) 24 h, methotrexate 20 mg PO q7days, and folic acid 10 mg PO q7d. After six months and clinical improvement the prednisone dose was adjusted (i.e. prednisone 7.5 mg PO q24 h) for two years. After withstanding a bout of purpuric pigmented dermatosis the dose of prednisone was adjusted to 15 mg PO q7d and topic clioquinol and clindamycin for two weeks was administered.

## 4 Clinical evolution and surgical management

Transesophageal echocardiography (TEE) was performed to assess presence aortic root abnormalities. The TEE reported a left ventricular ejection fraction of 66%, without the presence of intracavitary thrombi. Supra-aortic Doppler ultrasonography revealed a tardus-parvus waveform at the right vertebral artery suggestive of stenosis at the right subclavian artery. The right common carotid artery showed a hyperechoic image (i.e. 22 mm x 3 mm) compatible with an atheroma, without reduction of blood flow velocity (i.e. 20 cm/sec and 30% stenosis). An abdominal angiotomography was performed, reporting irregular wall thickening of the abdominal aorta with a 5.8 cm extension (i.e. originating below the emergence of the superior mesenteric artery and extending to the bifurcation of the iliac arteries) and both iliac arteries presented a total occlusion of approximately 4.3 cm (Fig. 1a and b). However, the arc of Riolan was permeable in its entirety (Fig. 1b).

Extra-anatomical revascularization was performed, with the placement of an 8 mm axillobifemoral poly-tetrafluoroethylene (PTFE)-based prosthetic graft. Sterile technique was procured at all times. The vascular access for the prosthetic graft placement was the left infra-clavicular region, with a right femoral end-to-end anastomosis. The graft was tunneled subcutaneously along the midaxillary line to prevent graft entanglement due to torso flexion. Upper and lower limb pulses were present after arterial unclamping. Minimal hemorrhage (i.e. less than 400cc) and no complications were reported during and after surgery. Seventy-two hours after the procedure the patient was discharged from the hospital with clinical improvement.

**Fig. 2:**
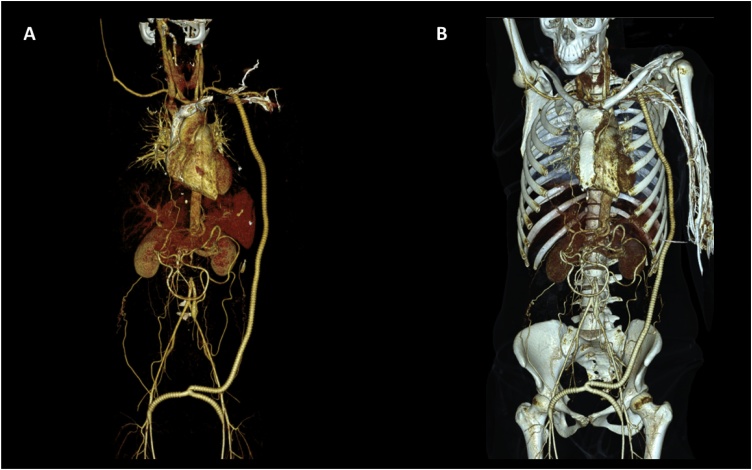
Post-surgical abdominal angiotomography showing a permeable axillobifemoral prosthetic graft. A and B) 3-D reconstruction computed tomography.

Two weeks after the surgical procedure the patient was clinically evaluated. The patient reported symptom improvement (i.e. paresthesia, dysesthesia, and claudication). Upon physical examination, both inferior limbs had femoral, popliteal, posterior tibial, and dorsal pedis pulses present, symmetric, rhythmic, with low amplitude, intensity ++/+++, and in synchrony with the heart rate. The skin showed no distal cyanosis, temperature changes or edema. The patient underwent pulsed-wave Doppler ultrasonography of the lower limbs with adequate blood flow. A post-surgical abdominal angiotomography was performed which showed a permeable axillobifemoral prosthetic graft (Fig. 2). The postoperative blood flow velocities are reported in Table 4. The patient was managed with prednisone 10 mg PO q24 h and rivaroxaban 20 mg PO q24 h, without reporting adverse effects (i.e. 3 months post-surgery).

**Table 4:** Post-operative blood flow velocities of major lower extremity arteries.

Anatomical region	Velocity (cm/sec)	
Lower limbs	Right	Left
Common femoral artery	44	44
Proximal segment superficial femoral artery	27	29
Middle segment superficial femoral artery	23	25
Distal segment superficial femoral artery	18	22
Popliteal artery	16	20
Proximal segment anterior tibial artery	14	19
Middle segment anterior tibial artery	10	13
Distal segment anterior tibial artery	9	11
Proximal segment posterior tibial artery	16	12
Middle segment posterior tibial artery	14	10
Distal segment posterior tibial artery	13	9
Proximal segment fibular artery	10	12
Middle segment fibular artery	8	9
Distal segment fibular artery	7	7

## 5 Discussion

We present the case report of a patient with TA, who after five years of corticosteroid and immunosuppressant management deteriorated clinically requiring surgical intervention with an axillobifemoral bypass for a total abdominal occlusion. Very limited literature exists regarding surgical interventions for TA patients. Most reported cases present endovascular surgical management. Although endovascular management is less invasive than extra-anatomical axillofemoral bypass, the patient was not a candidate for endovascular stent graft placement due to the increased risk for vascular injury and subsequent perforation. Considering the age of the patient, 22-years, open aortic repair is the preferred option and considering that axillobifemoral bypass surgery has the same long-term patency regardless of age, a strong argument can be made that this procedure should be avoided in younger populations. However, due to the patient’s overall fitness and after a preoperative assessment from the Internal Medicine and Anesthesiology departments a consensus was reached between these two departments and the Surgery department (i.e. the position of the surgical team was to perform an aortic repair) that open aortic repair was not a suitable procedure due to the high intraoperative risk. The left axillary artery was chosen as inflow vessel over the right even after having lower systolic blood pressure because the right subclavian artery had Doppler ultrasonography findings suggestive of stenosis and no distal aortic arch or left subclavian artery stenosis was detected through the same procedure. This case contributes to the existing literature with a successful surgical case that documented through various imaging modalities a patient for over five years.

Vascular surgery in TA cases becomes an option when the patient does not improve clinically after administration of medical treatment. Corticosteroids are the mainstay of the therapeutic management for TA, while immunosuppressant drugs (e.g. azathioprine, methotrexate, cyclophosphamide, mycophenolate, tacrolimus, and leflunomide) are widely used as maintenance therapy [7]. Furthermore, biopharmaceuticals such as infliximab and etanercept are therapeutic options for selected cases [12,13]. Over time about a fifth of the patients require surgical management and endovascular alternatives are preferred due to their less invasiveness. Percutaneous transluminal angioplasty and stent-graft placement are among the endovascular options available for TA, while surgical revascularization can be performed via surgical bypass grafting, patch angioplasty for short-segment lesions and endarterectomy [14].

Extra-anatomical axillofemoral bypass surgery is a procedure that is performed in other pathologies such as aortic coarctation, aortic aneurysm, aortoiliac occlusive disease, and TA. In 2004, a case similar to the one presented by our group successfully performed an axillobifemoral bypass for a total abdominal aorta occlusion [15]. Another example in the literature of successful axillobifemoral bypass in TA to treat both atypical coarctation and brachiocephalic involvement [16]. The case presented can be assessed as a successful procedure based on post-surgical clinical and imaging hemodynamic improvement. Although no intraoperative or perioperative complications were reported, these include brachial plexus injury, axillary pullout syndrome, graft thrombosis, delayed pseudoaneurysm of the graft, and graft infection. Clinically the patient had detectable bilateral lower extremity pulses, had reduced paresthesia and dysesthesia, as well as not reporting claudication during the three months after the surgical procedure. The patient also had increased post-surgical vascular velocities in both lower extremities, as well as an abdominal angiotomography with a permeable axillobifemoral prosthetic graft.

## 6 Limitations

Disease activity is a key factor that influences the decision to proceed with revascularization. Both endovascular and surgical revascularization should be avoided during acute TA disease activity as reocclusion and complications during the surgical procedure [17]. Disease activity is defined by a combination of clinical signs and symptoms, laboratory assessment and vascular imaging [7]. Both acute phase reactants, erythrocyte sedimentation rate, and C-reactive protein are useful to monitor TA disease activity; however, on their own, these serological tests prove to be insufficient to asses TA disease activity [18]. Although the patient did not have imaging or laboratory indicators for acute TA disease activity, active vasculitis cannot be completely dismissed. Among the two surgical approaches, open surgical intervention has a lower risk for restenosis than endovascular procedures as a late complication (i.e. 10-year follow-up), with a rate of restenosis of 37% versus 62%, respectively [6,[19], [20], [21]]. Another limitation of the study is the exclusion of other possible differential diagnoses. Middle aortic syndrome (MAS) could not be completely dismissed due to the patient’s young age. MAS can be due to congenital syndromes neurofibromatosis, mucopolysaccharidoses, Williams syndrome and, Alagille syndrome [22]. No genetic tests were performed on our patient, hence a genetic cause could be the etiology behind the aortic stenosis.

## 7 Conclusion

One in five TA patients over time become refractory to medical treatment. Vascular surgery is an option in these refractory cases. Two types of surgical interventions are available endovascular and open surgical procedures. Among the endovascular options available for TA are percutaneous transluminal angioplasty and stent-graft placement, while surgical revascularization can be performed via surgical bypass grafting, patch angioplasty for short-segment lesions and endarterectomy. Although endovascular management has fewer complications, the rate of restenosis is higher. Patients at risk of restenosis and who have increased perioperative vascular can benefit from open surgical procedures. The surgical management should be tailored to the patient’s needs, taking into account the extension of the lesion, available resources, and the treating surgeon’s experience.

## Declaration of Competing Interest

The authors declare that there are no conflicts of interest relevant to this work.

## Sources of funding

This study was supported by CONACyT (Consejo Nacional de Ciencia y Tecnología) Grant #440591 (Dr. Jaime Mondragón). This research did not receive any specific grant from funding agencies in the commercial sector.

## Ethical Approval

Approval from the ethical committee was not required due to the nature of this case report. Abiding by the Declaration of Helsinki, patient anonymity was guaranteed.

## Consent

Upon hospital admission, the patient signed an informed consent permitting the use of her clinical file information for didactic and research purposes.

## Author contribution

Study concept and design: OJZ, JDM

Acquisition of data: LNVR, AHR, MAL, MAMR, AMBB

Analysis and interpretation of data: OJZ, JDM, GAFS, PPA

Critical revision of the manuscript for important intellectual content: All authors.

All authors read and approved the final manuscript.

## Registration of Research Studies

All data necessary for the interpretation of this case is found in the text. No data depository or

registry was used.

## Guarantor

Jaime D. Mondragon and Omar Jiménez-Zarazúa

## Provenance and peer review

Not commissioned, externally peer-reviewed
